# A novel locus on mouse chromosome 7 that influences survival after infection with tick-borne encephalitis virus

**DOI:** 10.1186/s12868-018-0438-8

**Published:** 2018-07-06

**Authors:** Martin Palus, Yahya Sohrabi, Karl W. Broman, Hynek Strnad, Matyáš Šíma, Daniel Růžek, Valeriya Volkova, Martina Slapničková, Jarmila Vojtíšková, Lucie Mrázková, Jiří Salát, Marie Lipoldová

**Affiliations:** 1 Institute of Parasitology, Biology Centre of the Czech Academy of Sciences, Branišovská 31, 37005 České Budějovice, Czech Republic; 2 Department of Virology, Veterinary Research Institute, Hudcova 70, 62100 Brno, Czech Republic; 3 Department of Molecular and Cellular Immunology, Institute of Molecular Genetics, Academy of Sciences of the Czech Republic, Vídeňská 1083, 14220 Prague, Czech Republic; 4 Department of Biostatistics and Medical Informatics, 6770 Medical Sciences Center, 1300 University Avenue, Madison, WI 53706-1532 USA; 5 Department of Genomics and Bioinformatics, Institute of Molecular Genetics, Academy of Sciences of the Czech Republic, Vídeňská 1083, 14220 Prague, Czech Republic; 6 Department of Natural Sciences, Faculty of Biomedical Engineering, Czech Technical University in Prague, Sítná 3105, 272 01 Kladno, Czech Republic

**Keywords:** Tick-borne encephalitis virus (TBEV), Mouse model, Survival, Susceptibility locus, Chromosome 7, Candidate gene

## Abstract

**Background:**

Tick-borne encephalitis (TBE) is the main tick-borne viral infection in Eurasia. Its manifestations range from inapparent infections and fevers with complete recovery to debilitating or fatal encephalitis. The basis of this heterogeneity is largely unknown, but part of this variation is likely due to host genetic. We have previously found that BALB/c mice exhibit intermediate susceptibility to the infection of TBE virus (TBEV), STS mice are highly resistant, whereas the recombinant congenic strain CcS-11, carrying 12.5% of the STS genome on the background of the BALB/c genome is even more susceptible than BALB/c. Importantly, mouse orthologs of human TBE controlling genes *Oas1b, Cd209*, *Tlr3, Ccr5, Ifnl3* and *Il10*, are in CcS-11 localized on segments derived from the strain BALB/c, so they are identical in BALB/c and CcS-11. As they cannot be responsible for the phenotypic difference of the two strains, we searched for the responsible STS-derived gene-locus. Of course the STS-derived genes in CcS-11 may operate through regulating or epigenetically modifying these non-polymorphic genes of BALB/c origin.

**Methods:**

To determine the location of the STS genes responsible for susceptibility of CcS-11, we analyzed survival of TBEV-infected F_2_ hybrids between BALB/c and CcS-11. CcS-11 carries STS-derived segments on eight chromosomes. These were genotyped in the F_2_ hybrid mice and their linkage with survival was tested by binary trait interval mapping. We have sequenced genomes of BALB/c and STS using next generation sequencing and performed bioinformatics analysis of the chromosomal segment exhibiting linkage with TBEV survival.

**Results:**

Linkage analysis revealed a novel suggestive survival-controlling locus on chromosome 7 linked to marker D7Nds5 (44.2 Mb). Analysis of this locus for polymorphisms between BALB/c and STS that change RNA stability and genes’ functions led to detection of 9 potential candidate genes: *Cd33*, *Klk1b22*, *Siglece*, *Klk1b16*, *Fut2*, *Grwd1*, *Abcc6*, *Otog*, and *Mkrn3*. One of them, *Cd33*, carried a nonsense mutation in the STS strain.

**Conclusions:**

The robust genetic system of recombinant congenic strains of mice enabled detection of a novel suggestive locus on chromosome 7. This locus contains 9 candidate genes, which will be focus of future studies not only in mice but also in humans.

## Background

Tick-borne encephalitis (TBE) is the main tick-borne viral infection in Eurasia. It is prevalent across the entire continent from Japan to France [1]. The disease is caused by tick-borne encephalitis virus (TBEV), a flavivirus of the family *Flaviviridae*, which besides TBEV includes West Nile virus (WNV), dengue virus (DENV), Zika virus (ZIKV), yellow fever virus (YFV), Japanese encephalitis virus (JEV), and several other viruses causing extensive morbidity and mortality in humans. Ticks act as both the vector and reservoir for TBEV. The main hosts are small rodents, with humans being accidental hosts. In Europe and Russia between 5000 and 13,000 clinical cases of TBE are reported annually, with a large annual fluctuation [2]. The highest incidence of TBE is reported in western Siberia, in the Czech Republic, Estonia, Slovenia and Lithuania, but the prevalence of the disease is believed to be higher than actually reported [1, 2]. TBEV may produce a variety of clinical symptoms, from an asymptomatic disease to a fever and acute or chronic progressive encephalitis. The outcome of infection depends on the strain of virus [1], as well as on the genotype [3], sex and age of the host [4], and on the environmental and social factors [1]. Environmental and social factors influence also risk of infection.

Genetic influence on susceptibility to TBEV-induced disease has been analyzed by two main strategies: a hypothesis-independent phenotype-driven approach and a hypothesis-driven approach. Application of a genome-wide search (hypothesis-independent approach) in mouse led to identification of the gene *Oas1* (2′-5′-oligoadenylate synthetase gene) [5, 6]. A stop codon in exon 4 of the gene *Oas1b* (a natural knockout) present in majority of mouse laboratory strains causes production of protein lacking 30% of the C terminal sequence [5]. This part of molecule seems to be critical for tetramerization required for OAS1B activity leading to degradation of viral RNA. Thus, this mutation makes majority of mouse laboratory strains susceptible to flaviviruses [6, 7]. Human ortholog to this gene (*OAS1*) also modifies susceptibility to other flaviviruses (WNV) [8, 9], whereas *OAS2* and *OAS3* localized in the same cluster on chromosome 12q24.2 influence response to TBEV [3]. The polymorphic sites associated in *OAS2* and *OAS3* with susceptibility to TBEV did not resulted in amino acid changes, thus mechanisms of susceptibility control is not known [3]. The hypothesis-driven approach has focused on genes that encode molecules indicated to be involved in antiviral response by mechanistic studies [9]. These candidate genes studies revealed that polymorphisms in *CD209*/*DC*-*SIGN* [10], *CCR5* [11, 12], *TLR3* [12, 13], *IL10* [14] and *IFNL3*/*IL28B* [14] influence susceptibility to TBEV in humans.

**Fig. 1:**
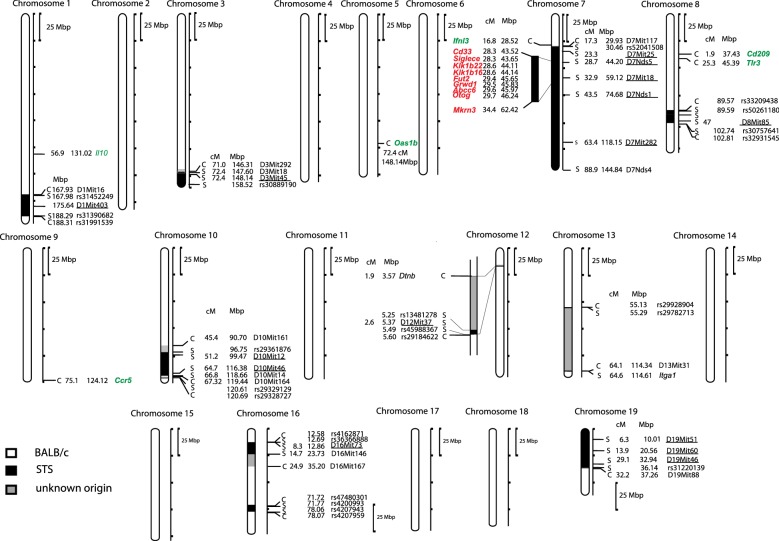
Genetic composition of the strain CcS-11. The regions of STS and BALB/c origin are represented as dark and white, respectively, the boundary regions of undetermined origin are shaded. Only the markers or SNPs defining the boundaries of STS-derived segment and markers that were tested for linkage (underlined) are shown. Genes *Oas1b, Cd209*, *Tlr3, Ccr5, Ifnl3* and *Il10,* known to control susceptibility to TBEV are shown in green, potential candidate genes *Cd33*, *Klk1b22*, *Siglece*, *Klk1b16*, *Fut2*, *Grwd1*, *Abcc6*, *Otog*, and *Mkrn3* detected in current study are shown in red

Our previous study has shown that both after subcutaneous and intracerebral inoculation of European prototypic TBEV, BALB/c mice exhibited intermediate susceptibility to the infection, STS mice were highly resistant, whereas the strain CcS-11, which carries 12.5% of the STS genome on the background of the genome of the strain BALB/c [15], is even more susceptible than its two parents—BALB/c and STS [16]. Importantly, mouse orthologs of human TBEV controlling genes: *Oas1b, Cd209*, *Tlr3, Ccr5, Il10* and *Ifnl3* are in CcS-11 localized on segments derived from the strain BALB/c (Fig. 1), so they are identical in both BALB/c and CcS-11 and hence cannot be responsible for the phenotypic difference of the two strains. Therefore, the difference must be due to a presently unknown locus, which could be detected by a linkage study of a cross between BALB/c and CcS-11. Thus, we have generated a F_2_ intercross between BALB/c and CcS-11 and performed a linkage and bioinformatics analysis. These studies revealed a novel suggestive locus on mouse chromosome 7 containing 9 potential candidate genes.

## Methods

### Mice

417 female F_2_ offspring of an intercross between strains CcS-11 and BALB/c (mean and median age 9.5 and 9 weeks, respectively, at the time of infection) were produced at the Institute of Molecular Genetics AS CR. Mice were tested in three successive experimental groups at the Institute of Parasitology, AS CR. When used for these experiments, strain CcS-11 had undergone more than 90 generations of inbreeding. Experiments Nr. 1, 2, and 3 comprised 120, 121 and 176 F_2_ mice, respectively. Sterilized pellet diet and water were supplied ad libitum. The mice were housed in plastic cages with wood-chip bedding, situated in a specific pathogen-free room with a constant temperature of 22 °C and a relative humidity of 65%.

### Virus infection and disease phenotype

Experiments were performed with European prototypic TBEV strain Neudoerfl (a generous gift from Professor F. X. Heinz, Medical University of Vienna). This strain was passaged five times in brains of suckling mice before the use in this study [16]. Mice were infected subcutaneously with 10^4^ pfu of the virus.

Mice were scored for mortality for a period of 35 days post-infection (p.i.) with TBEV, as well as presence of ruffled fur and paresis in three independent successive experiments at the Institute of Parasitology AS CR.

### Genotyping of F_2_ mice

DNA was isolated from tails using a standard proteinase procedure. The strain CcS-11 differs from BALB/c at STS-derived regions on eight chromosomes [17]. These differential regions were genotyped in the F_2_ hybrid mice between CcS-11 and BALB/c using 16 microsatellite markers (Generi Biotech, Hradec Králové, Czech Republic): D1Mit403, D3Mit45, D7Mit25, D7Nds5, D7Mit18, D7Nds1, D7Mit282, D7Mit259, D8Mit85, D10 Mit12, D10Mit46, D12Mit37, D16Mit73, D19Mit51, D19Mit60, D19Mit46 (Fig. 1) as described in [17].

### Statistical analysis

Survival, ruffled fur and paresis were treated as binary phenotypes (death/survival; presence/absence of symptom), and binary trait interval mapping was performed [18, 19]. A permutation test [20] was used to assess significance. This takes account of the limited genetic difference between the strains BALB/c and CcS-11. On the basis of 10,000 permutation replicates, the 5% significance LOD threshold was 2.56; the 10% threshold was 2.23. The Pearson correlation coefficient between presence of death and paresis was computed by the program Statistica for Windows 12.0 (StatSoft, Inc., Tulsa, OK).

### Detection of polymorphisms that change RNA stability and genes’ functions

We have sequenced the genomes of strains BALB/c and STS using next generation sequencing (NGS) system HiSeq 2500 (Illumina) (12× coverage). NGS data was preprocessed using software Trimmomatic [21] and overlapped paired reads were joined by software Flash [22]. Alignment—reference mouse sequence mm10 (build GRCm38)—was performed using BWA (Burrows-Wheeler Aligner) [23] program. Mapped reads were sorted and indexed, duplicated reads were marked. Segment covering peak of linkage on chromosome 7 from 36.2 to 74.5 Mb was inspected for polymorphisms between BALB/c and STS that change RNA stability and genes’ functions. Local realignment around indels, base recalibration and variants filtration were performed using software GATK (The Genome Analysis Toolkit) [24]. Variant annotation and effect prediction was performed by software SnpEff [25]. IGV (Integrated Genome Viewer) was used for visualization of results [26].

## Results

**Fig. 2:**
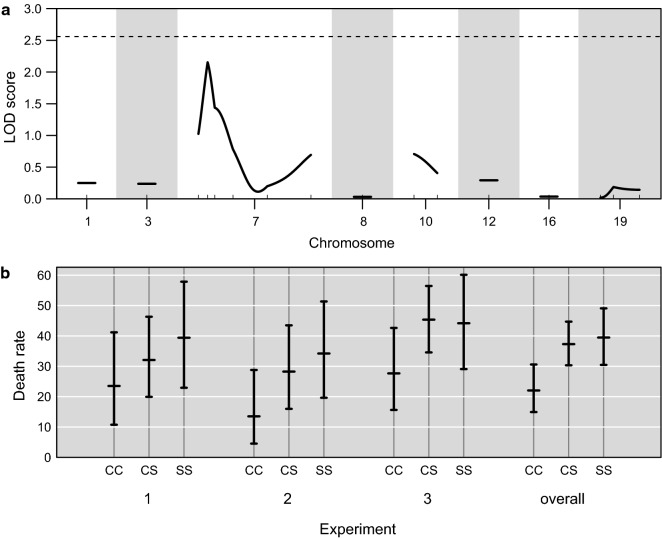
Genetic influence on susceptibility to TBEV in an F_2_ intercross between BALB/c and CcS-11. **a** LOD curves from binary trait interval mapping for death/survival. A dashed horizontal line is plotted at the 5% significance threshold, adjusting for the genome scan. **b** A plot of the death rate as a function of genotype at marker D7Nds5 and experiment, with 95% confidence intervals. C and S indicate the presence of BALB/c and STS allele, respectively. The S allele is associated with a higher death rate. The numbers of mice in the experiments 1, 2, and 3 were 120, 121 and 176, respectively

**Fig. 3:**
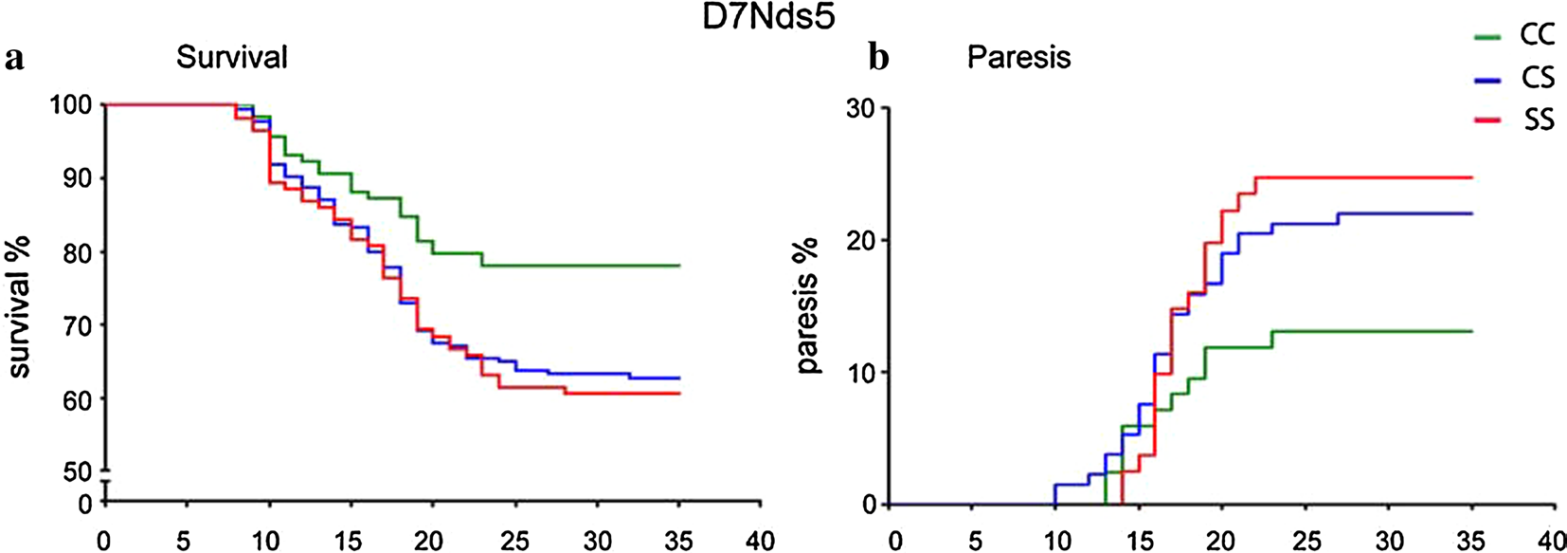
Differential survival (**a**) and incidence of paresis (**b**) of and in F_2_ hybrid mice carrying CC, CS and SS genotype at the marker D7Nds5 (n.s) after TBEV infection. Mice were infected subcutaneously with 10^4^ pfu of the TBEV strain Neudoerfl and observed for lethality for 35 days

Binary trait linkage analysis revealed a suggestive locus on chromosome 7 near D7Nds5 affecting the binary trait (death/survival) (LOD = 2.15), with a corresponding genome-scan-adjusted *P* value = 0.12 (Fig. 2a). The 1-LOD support interval spans from D7Mit25 to D7Nds1. The STS allele both in homozygotes and heterozygotes was associated with a higher death rate in each of the three separate experimental groups (Fig. 2b), and in the pooled data (Fig. 3a), so its presence in CcS-11 enhances even more the overall susceptibility determined by the BALB/c background. Ruffled fur was observed in only 8% of mice, so it was not suitable for statistical analysis. Paresis was less frequent than mortality (n = 60 vs. 102) and not all paretic mice died, but the two phenotypes were positively correlated (Pearson correlation 0.53). Moreover, frequency of paresis in the three D7Nds5 genotypes (Fig. 3b), although not significantly different, was biologically compatible with the survival data, because D7Nds5 CC homozygotes had the highest survival rate, and the lowest percentage of paresis.

We have sequenced genomes of BALB/c and STS and analyzed the segment covering peak of linkage on chromosome 7 from 36.2 to 74.5 Mb for polymorphisms between BALB/c and STS that change RNA stability and genes’ functions. This revealed 9 potential candidate genes: *Cd33* (CD33 antigen), *Klk1b22* (kallikrein 1-related peptidase b22), *Siglece* (sialic acid binding Ig-like lectin E), *Klk1b16* (kallikrein 1-related peptidase b16), *Fut2* (fucosyltransferase 2), *Grwd1* (glutamate-rich WD repeat containing 1), *Abcc6* (ATP-binding cassette, sub-family C (CFTR/MRP), member 6), *Otog* (otogelin), and *Mkrn3* (makorin, ring finger protein, 3) (Table 1, Fig. 1).

**Table 1:** List of candidate genes in TBEV susceptibility locus

Position Bp	Reference genotype C57BL/6	Genotype BALB/c	Genotype STS	Protein position of amino acid	Reference amino acid	Alteration	Type of change	Gene symbol	Transcription status	Gene name	Gene ID: MGI	Gene ID: NCBI
43,528,893	C/C	C/C	T/T	353	G	K	Single AA Change	*Cd33*	KNOWN	CD33 antigen	99,440	12,489
43,532,167	G/G	G/G	A/A	190	R	*	Nonsense Mutation	*Cd33*	KNOWN	CD33 antigen	99,440	12,489
43,659,827	G/G	G/G	T/T	102	D	E	Single AA Change	*Siglece*	KNOWN	Sialic acid binding Ig-like lectin E	1,932,475	83,382
44,115,970	A/A	A/A	C/A	115	L	Y	Single AA Change	*Klk1b22*	KNOWN	Kallikrein 1-related peptidase b22	95,291	13,646
44,140,534	G/G	G/G	C/C	76	G	A	Single AA Change	*Klk1b16*	KNOWN	Kallikrein 1-related peptidase b16	891,982	16,615
45,650,779	G/G	G/G	A/A	190	R	W	Single AA Change	*Fut2*	KNOWN	Fucosyltransferase 2	109,374	14,344
45,830,054	CTCTTCA/CTCTTCA	C/C	CTCTTCA/CTCTTCA	129	ED	.	Deletion	*Grwd1*	KNOWN	Glutamate-rich WD repeat containing 1	2,141,989	101,612
45,977,290	C/C	A/A	C/C	1448	V	L	Single AA Change	*Abcc6*	KNOWN	ATP-binding cassette, sub-family C (CFTR/MRP), member 6	1,351,634	27,421
46,262,804	C/C	C/C	T/T	748	R	W	Single AA Change	*Otog*	KNOWN	Otogelin	1,202,064	18,419
62,419,214	C/C	CGGCATTGGCACT/CGGCATTGGCACT	C/C	275	P	PVPMP	Insertion	*Mkrn3*	KNOWN	Makorin, ring finger protein, 3	2,181,178	22,652

Table shows differences between BALB/c and STS in DNA and protein sequences in potential candidate genes. Table shows also sequences of the reference mouse strain C57BL/6

One of these genes, *Cd33*, carried in the strain STS a nonsense mutation. Other changes in the strain STS in comparison with BALB/c (and the reference strain C57BL/6) represented single amino acids change in Siglec E, KLK1B22, KLK1B16, FUT2 and OTOG. The BALB/c strain had in comparison with STS (and the reference strain C57BL/6) deletion of two amino acids in GRWD1, insertion of four amino acids in MKRN3 and single amino acid change in and ABCC6 (Table 1).

## Discussion

CD33 and Siglec E belong to family of CD33-related sialic-acid-binding immunoglobulin-like lectins (CD33rSiglecs). They are ITIM-containing inhibitory receptors, which are involved in regulation of inflammatory and immune responses [27]. Gene *Cd33* carried in the strain STS a nonsense mutation (Table 1). Product of this gene is in mouse expressed on myeloid precursors and cells of myeloid origin [28] and on microglial cells [29]. It can inhibit response to amyloid plaques and its deletion leads to protection in the mouse model of Alzheimer disease (AD) [29] and in humans some *CD33* genetic variants are associated with late-onset AD [30]; its potential role in pathology of TBEV might be associated with its regulatory role in inflammatory responses. Gene *Siglece* carried in the strain STS a single amino acid change. Siglec E is expressed on microglia and inhibits neurotoxicity triggered by neural debris [31], which might have protective role against damage induced by flaviviruses.

A single amino acid change was present in KLK1B22 and KLK1B16. Kallikreins are serine proteases that might both help to fight infection by activating complement system [32], as well as aggravate disease symptoms by releasing bradykinin, which causes alterations in vascular permeability [33]. Their role in defense against flaviviruses has not been described. Kallikrein-bradykinin system have been described to contribute to protection against *Leishmania* [34] and *Trypanosoma cruzi* [35] parasites in mice. Interestingly, on the mouse chromosome 7 were in the strain CcS-11 mapped loci *Lmr21* and *Tbbr3* that control susceptibility to *L. major* [36] and *T. b. brucei* [17], respectively. However, both loci are mapped on a long chromosomal segment, thus other gene(s) might be responsible for their effect.

*FUT2* have been described to influence control of a wide range of pathogens such as noroviruses [37], rotaviruses [38], HIV [39], and *Escherichia coli* [40] in humans, and to *Helicobacter pylori* in mouse [41], but its role in resistance to flaviviruses is not known.

Makorin 1 induces degradation of WNV capsid which might protect host cells [42]. The E3 ligase domain responsible for MKRN1 effect is present also in MKRN3 [43]. Thus, gene *Mkrn3* might have relationship to defense against flaviviruses. Similarly, possible role of *Otog, Grwd1* and *Abcc6* in resistance to TBEV remains to be elucidated.

Public database BioGPS shows that all the candidate genes are in uninfected mice expressed in tissues such as brain, spleen and liver (Table 2). Brain is the main target for the virus; however, during the extraneural phase of the infection, several tissues and organs in the body are infected, including spleen and liver [44]. Highest expression in these tissues exhibits *Cd33* and *Siglece* with expression in microglia ten times higher than median value (> 10M), *Cd33* and *Klk1b22* are highly expressed in spleen (> 3M), > 10M expression of these two genes is also observed in bone marrow; *Siglece* is also highly expressed in bone (> 3M) and bone marrow macrophages (> 3M), whereas *Cd33* is highly expressed in granulocytes (> 30M), plasmacytoid dendritic cells (> 30M), osteoclasts (> 30M), myeloid dendritic cells (> 10M), in spleen (> 3M), lymph nodes (> 3M), eyecup (> 3M), B cells in marginal zone (> 3M) and in FoxP^+^ T cells (> 3M). For both *Klk1b22* and *Klk1b16* is characteristic very high expression in salivary gland (> 1000M) and high expression in lacrimal gland (> 30M). *Klk1b22* is also highly expressed in large intestine (> 30M), kidney (> 30M), pancreas (> 30M), testis (> 30M), stomach (> 10M), plasmacytoid dendritic cells (> 10M), small intestine (> 3M), spleen (> 3M) and CD8 + T cells (> 3M), whereas *Klk1b16* is also highly expressed in pituitary (> 30M), kidney (> 3M), pancreas (> 3M) and testis (> 30M). Highest expression of *Fut2* was observed in uterus (> 30M), and in stomach (> 10M), it was also highly expressed in large intestine (> 3M), prostate (> 3M) and in testis (> 3M). GRWD1 was described to play a role in ribosome biogenesis and during myeloid differentiation [45]. High expression level in hematopoietic stem cells (> 10M), mega erythrocyte progenitors (> 10M), granulocytes (> 10M), common myeloid progenitor (> 3M) supports this finding, but it is also expressed in several T cell subpopulations (> 3M), B cells in marginal zone (> 3M), as well as in lacrimal gland. *Abcc6* is highly expressed in liver (≫ 30M) and in lens (> 10M) and *Mrkn3* is highly expressed in retina (10M) and in olfactory bulb (> 3M). The expression data further support a potential role of detected candidate genes in defense against TBEV, but they must be in the future complemented with data describing gene expression after TBEV infection.

**Table 2:** Expression of potential candidate genes in organs and cells of uninfected mice

Gene symbol	*Cd33*		*Siglece*		*Klk1b22*		*Klk1b16*		*Fut2*		*Grwd1*		*Abcc6*		*Otog*		*Mkrn3*	
*Gene ID: MGI*	99,440		1,932,475		95,291		891,982		109,374		2,141,989		1,351,634		1,202,064		2,181,178	
*Gene ID: NCBI*	12,489		83,382		13,646		16,615		14,344		101,612		27,421		18,419		22,652	
*Median*	5.2		4.7		4.6		4.6		4.9		163		4.6		4.95		4.8	
***Organs***																		
Bone	77.05	> 10M	20.82	> 3M	4.64	~ M	4.64	~ M	4.86	~ M	86.89	<M	4.64	~ M	4.95	~ M	4.64	~ M
Bone marrow	114.05	> 10M	51.63	> 10M	4.64	~ M	4.64	~ M	4.88	~ M	106.44	~ M	4.64	~ M	4.79	~ M	4.66	~ M
Brain																		
Amygdala	4.97	~ M	4.67	~ M	4.64	~ M	4.64	~ M	4.86	~ M	73.20	<M	4.64	~ M	4.95	~ M	4.81	~ M
Cerebellum	4.91	~ M	4.76	~ M	5.24	~ M	5.64	~ M	5.39	~ M	26.75	<M	4.64	~ M	4.94	~ M	5.86	~ M
Cerebral cortex	4.91	~ M	4.67	~ M	4.64	~ M	4.64	~ M	4.90	~ M	93.30	<M	4.64	~ M	4.94	~ M	4.64	~ M
Hippocampus	4.91	~ M	4.67	~ M	4.64	~ M	4.64	~ M	4.86	~ M	51.91	<M	4.64	~ M	4.95	~ M	4.76	~ M
Olfactory bulb	4.89	~ M	4.67	~ M	4.64	~ M	4.67	~ M	4.86	~ M	38.09	<M	4.64	~ M	4.95	~ M	19.91	> 3M
Eye																		
Eyecup	13.71	~ 3M	4.67	~ M	6.64	> M	4.64	~ M	5.80	~ M	180.25	~ M	4.64	~ M	4.95	~ M	7.49	> M
Lens	9.26	> M	6.18	~ M	4.64	~ M	7.54	> M	5.80	~ M	392.8	> M	56.53	~ 10M	4.95	~ M	13.36	> M
Retina	5.72	~ M	4.67	~ M	4.74	~ M	4.64	~ M	4.86	~ M	147.73	<M	4.64	~ M	4.95	~ M	38.78	~ 10M
Intestine large	4.86	<M	4.67	~ M	486.82	≫ 30M	6.13	> M	91.36	> 3M	62.6	<M	4.64	~ M	4.94	~ M	4.64	~ M
Intestine small	4.91	~ M	4.68	~ M	35.44	> 3M	4.66	~ M	6.45	~ M	128.54	~ M	5.80	> M	4.95	~ M	4.64	~ M
Kidney	4.87	~ M	4.67	~ M	607.67	> 30M	15.57	~ 3M	10.44	> M	80.87	<M	4.64	~ M	4.95	~ M	4.64	~ M
Lacrimal gland	4.92	~ M	4.67	~ M	541.25	> 30M	187.21	> 30M	15.28	> M	286.48	> 3M	4.64	~ M	4.95	~ M	4.64	~ M
Liver	4.89	~ M	5.09	~ M	4.64	~ M	6.14	> M	4.86	~ M	161.71	~ M	920.68	≫ 30M	4.95	~ M	4.64	~ M
Lymph nodes	85.8	> 3M	4.68	~ M	4.64	~ M	4.64	~ M	4.99	~ M	97.85	<M	4.64	~ M	4.95	~ M	4.64	~ M
Pancreas	7.40	~ M	5.15	~ M	1806.5	> 30M	22.47	> 3M	10.44	> M	25.91	~ M	4.64	~ M	9.28	> M	4.64	~ M
Pituitary	4.89	~ M	4.67	~ M	13.10	~ 3M	114.09	~ 30M	7.07	> M	61.18	<M	4.64	~ M	4.95	~ M	4.64	~ M
Prostate	5.25	~ M	4.75	~ M	4.64	~ M	4.66	~ M	42.15	> 3M	48.62	<M	4.64	~ M	4.95	~ M	4.64	~ M
Salivary gland	5.62	~ M	4.67	~ M	36,542.06	> 1000M	26,974.63	> 1000M	5.95	~ M	189.83	~ M	4.64	~ M	4.99	~ M	5.25	~ M
Spleen	15.39	~ 3M	9.02	> M	13.11	~ 3M	4.64	~ M	4.91	~ M	122.09	<M	4.64	~ M	4.95	~ M	4.81	~ M
Stomach	4.89	~ M	4.65	~ M	81.39	> 10M	4.64	~ M	117.61	> 10M	73.50	<M	4.64	~ M	4.95	~ M	4.64	~ M
Testis	5.40	~ M	4.67	~ M	567.00	> 30M	30.89	> 3M	16.28	~ 3M	86.24	<M	10.55	> M	4.95	~ M	6.04	~ M
Uterus	4.91	~ M	4.67	~ M	4.64	~ M	4.64	~ M	222.61	> 30M	131.6	<M	4.64	~ M	4.95	~ M	5.84	~ M
***Cells***																		
B cells_marginal_zone	25.26	> 3M	4.67	~ M	4.97	~ M	4.64	~ M	4.86	~ M	446.31	~ 3M	4.64	~ M	4.95	~ M	4.64	~ M
Common myeloid progenitor	11.3	> M	6.18	~ M	5.77	~ M	5.05	~ M	5.20	~ M	1192.34	> 3M	4.64	~ M	5.91	~ M	4.64	~ M
Dendritic lymphoid cells	9.83	> M	4.67	~ M	4.64	~ M	4.64	~ M	4.86	~ M	369.07	> M	4.64	~ M	5.11	~ M	4.64	~ M
Dendritic cells myeloid CD8a−	77.51	> 10M	4.96	~ M	4.64	~ M	4.64	~ M	4.86	~ M	299.19	> M	4.64	~ M	4.95	~ M	4.64	~ M
Dendritic plasmacytoid B220+	140.59	~ 30M	4.74	~ M	88.52	> 10M	4.64	~ M	4.86	~ M	238.95	> M	4.64	~ M	5.14	~ M	4.64	~ M
Granul ocytes mac1 + gr1+	651.27	> 30M	6.87	> M	4.64	~ M	4.64	~ M	5.04	~ M	53.79	<M	4.64	~ M	4.95	~ M	4.64	~ M
Hematopoietic stem cells	42.14	> 3M	4.8	~ M	4.96	~ M	4.64	~ M	6.41	> M	1445.80	~ 10M	4.64	~ M	4.95	~ M	5.19	~ M
Macrophage_bone_marrow	11.64	> M	14.68	> 3M	4.64	~ M	4.64	~ M	4.86	~ M	308.83	> M	4.64	~ M	4.95	~ M	4.64	~ M
Mast cells	214.54	> M	4.67	~ M	4.64	~ M	4.64	~ M	4.83	~ M	55.07	<M	4.64	~ M	4.95	~ M	4.64	~ M
Mega_erythrocyte progenitor	5.31	~ M	4.67	~ M	7.57	> M	4.64	~ M	6.47	> M	2842.61	> 10M	4.64	~ M	8.07	> M	4.64	~ M
Microglia	99.81	> 10M	79.64	> 10M	4.64	~ M	4.64	~ M	4.86	~ M	269.08	> M	4.64	~ M	4.95	~ M	4.64	~ M
Osteoclasts	363.46	> 30M	4.77	~ M	7.67	> M	4.64	~ M	4.99	~ M	327.07	> M	4.64	~ M	5.31	~ M	4.64	~ M
T-cellsCD4+	4.91	~ M	5.00	~ M	7.57	> M	4.82	~ M	4.86	~ M	621.74	> 3M	4.64	~ M	5.06	~ M	4.64	~ M
T-cellsCD8+	4.91	~ M	4.67	~ M	13.55	~ 3M	4.64	~ M	5.29	~ M	715.19	> 3M	4.64	~ M	6.13	> M	4.64	~ M
T-cells FoxP3+	25.67	> 3M	4.67	~ M	4.64	~ M	4.96	~ M	4.93	~ M	584.63	~ 3M	4.64	~ M	4.95	~ M	4.64	~ M

Data were compiled from public database BioGps (http://biogps.org) May 6, 2018. First column: relative units; second column: relationship to median (M); M = median value across all samples for a single probe set

We have found a susceptibility allele of a locus on chromosome 7 in the resistant strain STS. This apparent paradox is likely caused by the fact that most inbred mouse strains were produced without an intentional selective breeding for a specific quantitative phenotype (like susceptibility to specific infections). Therefore they inherited randomly from their non-inbred ancestors susceptible alleles at some loci and resistant alleles at others, so that their overall susceptibility phenotype depends on the relative number of both types of alleles. Such finding is not unique, as susceptibility alleles originating from resistant strains were found in susceptibility studies of other infectious diseases [17, 46, 47] and colon cancer [48]. Similarly, in different in vitro tests of immune responses a low-responder allele was identified in a high responding strain [49] or vice versa [50]. Another explanation might be presence of BALB/c allele interacting with STS allele on chromosome 7. Demonstration of such interaction would require further experiments. We have already observed interaction of STS and BALB/c alleles leading to extreme phenotypes in susceptibility to *L. major* [51] and *L. tropica* [47].

## Conclusion

Mapping of TBEV controlling genes in mice is not easy due to presence of a strong TBEV controlling gene *Oas1b,* which is identical both in BALB/c and CcS-11, as well as in majority of laboratory mouse strains [6, 7] and masks effects of other controlling genes. Therefore using a powerful genetic system—recombinant congenic strains, we succeeded in mapping novel TBEV susceptibility locus on chromosome 7 and identified 9 potential candidate genes. Products of some of these genes have been described to participate in defense against flaviviruses, the role of the others is unknown. The genes detected here will be focus of future studies that will include characterization of candidate gene(s) products in BALB/c and CcS-11, introducing modification to candidate genes and study their influence on disease outcome in mouse, and study influence of polymorphisms in human orthologs of candidate genes on susceptibility to TBEV in humans.

## List of abbreviations

*Abcc6* (ATP-binding cassette, sub-family C (CFTR/MRP), member 6), mouse gene; *ABCC6*-human gene; ABCC6-protein (gene product); *Ccr5:* chemokine (C–C motif) receptor 5*; Cd209:* CD209 antigen; *Cd33* (CD33 antigen); *Fut2* (fucosyltransferase 2); *Grwd1* (glutamate-rich WD repeat containing 1); *Ifnl3:* interferon lambda 3 (synonym *Il28b*); *Il10*: interleukin 10; *Klk1b16* (kallikrein 1-related peptidase b16); *Klk1b22* (kallikrein 1-related peptidase b22); *Mkrn3* (makorin, ring finger protein, 3); *Oas1b:* 2′-5′-oligoadenylate synthetase gene; *Otog* (otogelin); pfu: Plaque-forming unit; RC: Recombinant congenic; s.c: Subcutaneous; *Siglece* (sialic acid binding Ig-like lectin E); TBE: Tick-borne encephalitis; TBEV: Tick-borne encephalitis virus; *Tlr3:* toll-like receptor 3.

According to current gene and protein nomenclature, mouse gene symbols are italicized, with only the first letter in upper-case (e.g. *Cd33*). Protein symbols are not italicized, and all letters are in upper-case (e.g. CD33). Human gene symbols are in upper-case and are italicized (e.g. *CD33*). Protein symbols are identical to their corresponding gene symbols except that they are not italicized (e.g. CD33).
